# Enhanced OCT4 transcriptional activity substitutes for exogenous SOX2 in cellular reprogramming

**DOI:** 10.1038/srep19415

**Published:** 2016-01-14

**Authors:** Adele G. Marthaler, Kenjiro Adachi, Ulf Tiemann, Guangming Wu, Davood Sabour, Sergiy Velychko, Ingo Kleiter, Hans R. Schöler, Natalia Tapia

**Affiliations:** 1 Department of Cell and Developmental Biology, Max Planck Institute for Molecular Biomedicine, Röntgenstraße 20, 48149 Münster, Germany; 2 Medical Faculty, Heinrich Heine University, Moorenstraße 5, 40225 Düsseldorf, Germany; 3 Department of Neurology, St Josef Hospital, Ruhr University Bochum, Gudrunstraße 56, 44791 Bochum, Germany; 4 Medical Faculty, University of Münster, Domagkstraße 3, 48149 Münster, Germany

## Abstract

Adenoviral early region 1A (E1A) is a viral gene that can promote cellular proliferation and de-differentiation in mammalian cells, features required for the reprogramming of somatic cells to a pluripotent state. E1A has been shown to interact with OCT4, and as a consequence, to increase OCT4 transcriptional activity. Indeed, E1A and OCT4 are sufficient to revert neuroepithelial hybrids to pluripotency, as demonstrated in previous cell fusion experiments. However, the role that E1A might play in the generation of induced pluripotent stem cells (iPSCs) has not been investigated yet. In this report, we show that E1A can generate iPSCs in combination with OCT4 and KLF4, thus replacing exogenous SOX2. The generated iPSCs are *bona fide* pluripotent cells as shown by *in vitro* and *in vivo* tests. Overall, our study suggests that E1A might replace SOX2 through enhancing OCT4 transcriptional activity at the early stages of reprogramming.

Somatic cells can acquire a pluripotent fate after ectopic expression of *Oct4, Sox2, Klf4,* and *Myc*^1^, from which *Myc* can be omitted^2^. Although OCT4 and SOX2 together with NANOG co-occupy the core targets of the pluripotency transcriptional network in embryonic stem cells (ESCs)^3^, *Nanog* is not essential to maintain pluripotency^4^. In contrast, repression of *Oct4* or *Sox2* in ESCs promotes differentiation^5^,^6^. OCT4 and SOX2 proteins dimerize on DNA to activate their target genes in order to specify pluripotency^7^. Interestingly, single-point mutations introduced in the SOX2-OCT4 interacting surface are sufficient to prevent their cooperative binding, which in turn precludes the maintenance and induction of pluripotency^8^,^9^.

In differentiated cells, OCT4 can activate gene transcription when its DNA binding site is located close to the TATA box. However, OCT4 requires additional factors to induce transcription when the DNA binding sites are distantly located from the TATA element. In fact, a remotely bound OCT4 requires coactivators for bridging the distance between the OCT4 binding site and the TATA box^10^. The adenovirus early region 1A (E1A) protein can function as such a coactivator and can enhance OCT4 transcriptional activity in somatic cells^10^. E1A does not directly bind to DNA, instead E1A interacts with components of the general transcriptional machinery such as the TATA-binding proteins and to several transcription factors. Indeed, the conserved domain 3 of the E1A protein has been described to bind to the OCT4 C-terminal domain^10^. Furthermore, Hamada and colleagues demonstrated that the combined expression of OCT4 and E1A is sufficient to revert neuroepithelial-like cell hybrids to pluripotent embryonic carcinoma-like cells in cell fusion experiments^11^. Taking together, E1A can enhance OCT4 transactivation activity in somatic cells and both proteins can together reprogram differentiated cells in a cell fusion context. Therefore, we sought to investigate the potential role of E1A in reprogramming somatic cells into induced pluripotent stem cells (iPSCs).

## Results

### *E1A* can replace exogenous *Sox2* in iPSCs reprogramming

**Figure 1:**
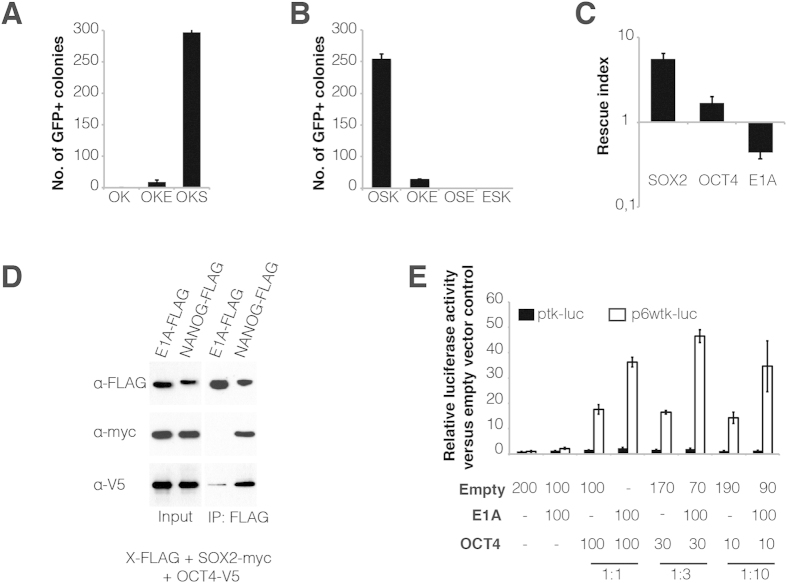
*E1A* can replace exogenous *Sox2* in reprogramming MEFs into iPSCs. (**A**) Average number of *Oct4*-GFP—positive (GFP+) iPSC colonies generated from MEFs after transduction with different combinations of *Oct4* (O), *Klf4* (K), *E1A* (E), and *Sox2* (S). Colonies were counted at day 22 after viral transduction. Error bars represent standard deviation of the mean of 5 biological replicates. (**B**) Average number of *Oct4*-GFP—positive (GFP+) iPSC colonies as described in (**A**). Colonies were counted at day 19 after viral transduction. Error bars represent standard deviation of the mean of 2 biological replicates. (**C**) *Sox2*-null ESCs were transfected with hygromycin-resistant PiggyBac plasmids coding for *Sox2, Oct4*, or *E1A*, and the empty vector as a negative control. After 1 week in culture in the presence of doxycycline (1 μg/ml) and hygromycin (300 μg/ml), AP staining was performed to quantify the number of surviving ESC colonies and the number of AP—positive colonies per cm^2^ was counted. The rescue index was calculated by dividing the number of AP—positive colonies for each construct by the number of AP—positive colonies in the empty PiggyBac vector. Error bars represent standard deviation of the mean of 6 biological replicates. (**D**) 293T cells were transfected with expression vectors encoding the gene of interest coupled to an epitope tag: *E1A-*FLAG plus *Sox2*-myc and *Oct4*-V5. *Nanog*-FLAG plus *Sox2*-myc and *Oct4*-V5 served as a positive control. Immunoprecipitated E1A- or NANOG-FLAG and co-immunoprecipitated SOX2-myc and OCT4-V5 are shown by Western blot. Input corresponds to 2% of the cell lysis extract. (**E**) The transactivation activity of OCT4 in the presence or absence of E1A was evaluated using the 6wtk luciferase reporter and plotted relative to the levels of the empty vector control. Error bars reflect standard errors (biological replicates: n = 3).

As E1A have been described to reprogram neuroepithelial-like cells into pluripotency^11^, we assessed whether *E1A* could also contribute to iPSC formation. Mouse embryonic fibroblasts (MEFs) containing an *Oct4*-green fluorescent protein (GFP) reporter construct were transduced using several combinations of retroviral-defective particles coding for *Oct4* (O), *Sox2* (S), *Klf4* (K), and *E1A* (E) (Fig. 1A). MEFs could not be reprogrammed into iPSCs using OK alone, as shown by the lack of *Oct4*-GFP—positive colonies. However the addition of *E1A* to OK led to the formation of iPSC colonies (Fig. 1A), indicating that E1A could replace exogenous SOX2. Interestingly, E1A could only substitute for exogenous SOX2 and not for exogenous OCT4 or KLF4 in iPSC induction (Fig. 1B).

Next, we sought to investigate the role of E1A during reprogramming. To this end, we assessed whether E1A could substitute exogenous SOX2 through the activation of OCT4-SOX2 target genes using *Sox2*-null ESCs, in which *Sox2* is expressed from a transgene that can be repressed upon doxycycline induction^5^. Hygromycin-resistant PiggyBac vectors coding for SOX2, OCT4, E1A and the empty vector were transfected into *Sox2*-null ESCs. One week after addition of hygromycin and doxycycline, the rescue index that corresponds to the number of alkaline phosphatase (AP)—positive colonies in the presence of each construct divided by the number of AP—positive colonies in the presence of the empty PiggyBac vector was calculated. Our results show that E1A could not rescue *Sox2*-null ESC self-renewal (Fig. 1C). Furthermore, *Sox2*-null ESC lines could be established from *Sox2*-null ESCs transfected with OCT4 and SOX2 but not with E1A and the empty vector (data not shown). These results demonstrate that E1A does not replace exogenous SOX2 through the activation of the OCT4-SOX2 target genes. We verified OCT4-E1A interaction using a co-immunoprecipitation experiment in which E1A-FLAG was overexpressed in the presence of SOX2-myc and OCT4-V5. NANOG-FLAG was used as a positive control for a double OCT4-SOX2 interaction. After pulling-down with an anti-FLAG antibody, we could demonstrate E1A interaction with OCT4 but not with SOX2 (Fig. 1D). Finally, we assessed whether E1A could enhance OCT4 transcriptional activity using a luciferase assay. A previous report has demonstrated that a certain ratio of OCT4 and E1A is necessary for efficient activation of a luciferase reporter containing POU octamer motifs^10^, thus we tested three different OCT4:E1A ratios. Our results show an increased transcriptional enhancement by OCT4 in the presence of E1A in all three conditions. Therefore, we conclude that E1A is able to interact with OCT4 and substitute for exogenous SOX2 in iPSC reprogramming, most likely, through the enhancement of OCT4 transcriptional activity^10^.

### iPSCs generated with exogenous E1A are fully pluripotent

**Figure 2:**
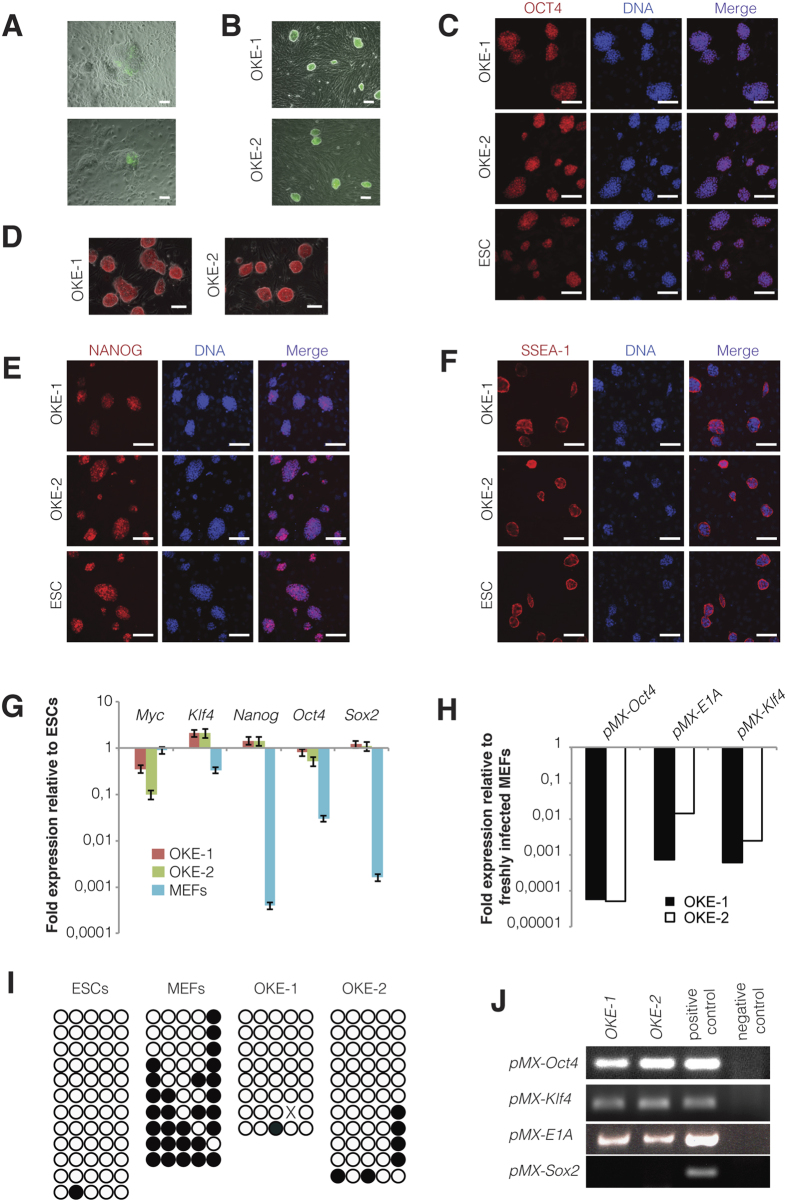
Molecular characterization of OKE iPSCs. (**A**) OKE GFP—positive cells observed 15 days after viral transduction. Scale bars, 100 μm. (**B**) *Oct4*-GFP expression in two stable derived OKE cell lines at passage 3. Scale bars, 100 μm. (**C**) OCT4, (**D**) AP, (**E**) NANOG, and (**F**) SSEA-1 immunofluorescence staining images of OKE-1 and -2 clones at passage 5. Scale bars, 100 μm. (**G**) Expression levels of pluripotency genes measured by qRT-PCR at passage 5. Data were plotted relative to ESCs. Error bars indicate standard deviation of calculations based on ΔΔCt-values obtained from two housekeeping genes, *Actb* and *Rpl37a*. (**H**) Silencing of the retroviral vectors shown by qRT-PCR. OKE-1 and OKE-2 were analyzed at passage 24 and 14, respectively. Data were normalized to *Hprt* and plotted relative to freshly transduced MEFs. (**I**) Methylation analysis of the *Oct4* promoter in OKE iPSCs passage 5 compared with ESCs and MEFs. Open and filled circles represent unmethylated and methylated CpGs, respectively. X represents an undetermined CpG. (**J**) PCR genotyping to detect retroviral insertions in OKE-1 and -2 iPSC clones at passage 5. Freshly transduced MEFs and wild-type ESCs were used as a positive and negative control, respectively. As expected, OKE-1 and -2 iPSCs do not contain the *Sox2* exogenous transgene.

**Figure 3:**
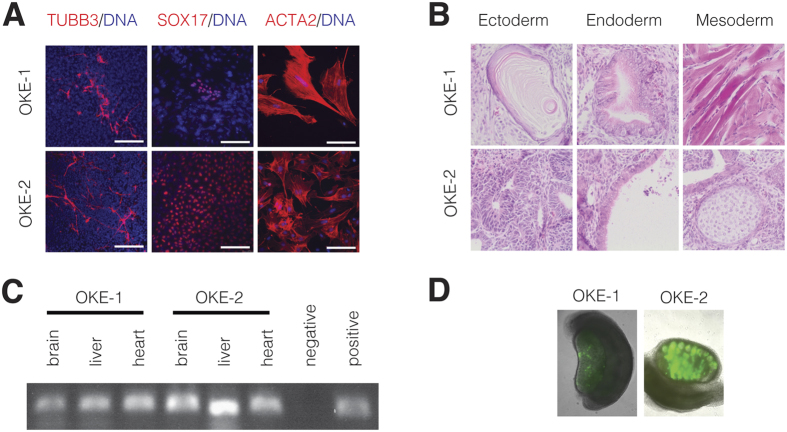
Differentiation potential of OKE iPSCs. (**A**) Immunocytochemistry for marker proteins representative of the three germ layers, TUBB3 (ectoderm), SOX17 (endoderm), and ACTA2 (mesoderm), after *in vitro* differentiation of OKE iPSCs (passage 10) by embryoid body formation. Scale bars, 150 μm. (**B**) Teratomas generated by OKE iPSC clones at passage 5 contained tissues of all three germ layers: ectoderm (keratinocyte and neural rosettes), endoderm (gut-like endothelium), and mesoderm (muscle and cartilage). (**C**) GFP-PCR genotyping to detect contribution of OKE-1 and -2 iPSCs in chimeric animals to organs of all three germ layers: brain (ectoderm), liver (endoderm), and heart (mesoderm). ESCs containing an *Oct4*-GFP construct and non-transgenic MEFs served as positive and negative controls, respectively. (**D**) Germline contribution visualized by *Oct4*-GFP—positive cells in a female and a male 13.5 days post-coitum embryonic gonad.

Next, we sought to verify the pluripotent potential of MEF-derived iPSCs generated with the novel OKE reprogramming cocktail. Two OKE iPSC clones, OKE-1 and OKE-2, were picked (Fig. 2A), expanded and analyzed. Both clones expressed the *Oct4*-GFP reporter (Fig. 2B) and stained positive for the pluripotency-markers alkaline phosphatase, OCT4, NANOG, and SSEA-1 (Fig. 2C–F). Additionally, endogenous *Oct4, Nanog, Sox2, Klf4* and *Myc* were expressed at levels similar to those of ESCs (Fig. 2G), while the exogenous factors were effectively silenced (Fig. 2H), as shown by quantitative RT-PCR. OKE iPSCs displayed an unmethylated *Oct4* promoter, characteristic for pluripotent cells (Fig. 2I). PCR genotyping demonstrated the presence of the OKE transgenes and confirmed the absence of exogenous *Sox2* (Fig. 2J). OKE-1 and OKE-2 were propagated *in vitro* for 30 passages, probing the stability of the new acquired cell fate identity. The differentiation potential of iPSCs *in vitro* was investigated by embryoid body formation, which was generated using the hanging-drop method. After 2 weeks of differentiation, specific cell types for ectoderm (TUBB3), endoderm (SOX17), and mesoderm (ACTA2) were detected by immunofluorescence microscopy (Fig. 3A). The *in vivo* differentiation potential was evaluated by teratoma formation. After 6–8 weeks of subcutaneous injection of iPSC lines into nude athymic mice, teratomas containing tissues of all three germ layers were observed (Fig. 3B). Additionally, OKE iPSCs were aggregated with mouse morula-stage embryos and were able to contribute to all germ layers as shown by PCR-genotyping for the GFP transgene (Fig. 3C). Germline contribution was observed by the presence of *Oct4*-GFP—positive cells in the gonads of 13.5-days post-coitum female and male embryos (Fig. 3D). Taken together, our data indicates that OKE iPSC lines exhibit the capacity to differentiate into cells of all three germ layers. In summary, we generated *bona fide* iPSCs with the viral factor *E1A* in combination with *Oct4* and *Klf4*, in the absence of exogenous *Sox2*.

## Discussion

OCT4 and SOX2 constitute the core of the pluripotency network and cooperatively bind and activate their target genes^3^. Indeed, *Sox2*-null ESCs differentiate into trophectoderm after SOX2 depletion, demonstrating that SOX2 is required to maintain pluripotency^5^. Interestingly, *Sox2*-null differentiation can be rescued upon *Oct4* overexpression, suggesting that the main role of SOX2 is to sustain OCT4 expression^5^. In line with these results, we speculate that E1A can substitute exogenous SOX2 during iPSC reprogramming due to their ability to enhance OCT4 transcriptional activity^10^. OCT4-rescued *Sox2*-null ESCs express a subset of *Oct*-*Sox* enhancer genes, such as *Utf1* or *Fgf4*, at similar levels than ESCs^5^. Based on these findings Masui and colleagues concluded that, in the presence of sufficient levels of OCT4, SOX2 is not required to activate a subset of genes containing *Oct*-*Sox* elements^5^, most of which are key genes in the pluripotency network^3^. E1A and OCT4 alone cannot activate *Oct*-*Sox* enhancers (e.g. *Fgf4*)^12^, an observation that is also demonstrated by the inability of E1A to rescue *Sox2*-null ESCs (Fig. 1C). Thus, our results point to E1A’s bridging capacity between OCT4 and the basal transcriptional machinery as the main function underlying its reprogramming potential. However, OKE reprogramming efficiency is much lower than OKS, suggesting that SOX2 can enhance OCT4 transcriptional activity more strongly than E1A or that SOX2 can play other roles that cannot be replaced by E1A.

The molecular events leading to the reprogramming of somatic cells into iPSCs remain unknown. However, the available data supports a model with a stochastic phase followed by a hierarchical phase^13^. In the initial phase, the exogenous factors are overexpressed at non-physiological levels, a scenario that promotes nonspecific binding to low-affinity binding sites and generates a transient transcriptionally unstable state^14^. In the final phase, some cells are able to activate endogenous pluripotent genes, inducing a hierarchical cascade of events that finally leads to iPSC generation. In this model, endogenous *Sox2* has been proposed to be the upstream gene that activates the endogenous pluripotency program at the late stage of iPSC induction^13^. In addition, experiments in which each of the four exogenous factors is added sequentially to the cells have demonstrated that the sequence ending with the addition of exogenous *Sox2* improves the reprogramming efficiency^15^. All these evidences point to exogenous SOX2 not being essential to launch the reprogramming program. Thus we hypothesize that reprogramming initiation requires enhanced OCT4 transcriptional activity, which can proceed in the absence of exogenous SOX2, but that reprogramming termination depends on endogenous SOX2. Further studies are required to determine at which time point of the iPSC generation needs endogenous *Sox2* to be activated. In summary, our study provides a new insight into the reprogramming mechanism and demonstrates that E1A can replace exogenous SOX2 due to the enhancement of OCT4 transcriptional activity, most likely by augmenting its interaction with the basal transcription machinery.

## Methods

### Cell Lines

MEFs were derived from mice containing a GFP transgene driven by the *Oct4* promoter^16^. Inducible *Sox2*-null ESCs carrying the transgenes tet-off *Sox2*, a tet transactivator, and a constitutive red-fluorescent protein transgene (DsRed) have been previously described^5^. Mouse experiments were performed in accordance with the recommendations of the Federation of Laboratory Animal Science Associations (FELASA) and were approved by the Landesamt für Natur, Umwelt und Verbraucherschutz (LANUV) of the state of North Rhine-Westphalia (Germany).

### Cell Culture Conditions

MEFs were cultured in DMEM medium, supplemented with 10% fetal bovine serum. ESC medium was used for all pluripotent cell lines and consisted of Knockout DMEM supplemented with 20% Knockout serum replacement, 0.2× ß-Mercaptoethanol, 1× nonessential amino acids and 1× penicillin/streptomycin/glutamine, and 1,000 U/ml leukemia inhibitory factor (in house preparation).

### Generation and characterization of iPSCs

pMX retroviral plasmids coding for *Oct4* (Addgene # 13366), *Klf4* (Addgene # 13370), *Sox2* (Addgene # 13367), *Myc* (Addgene # 13375)^1^, *E1A* (lacking the N-terminus and conserved region 1) (cloned in house) were cotransfected with the pCL-ECO packaging plasmid (Addgene # 12371)^17^ into 293T cells using Fugene®. Two days later, the supernatants were collected and filtered. MEFs were plated one day before transduction on gelatin-coated 6-well plates (5 × 10^4^ cells/well) and were transduced with 500 μl of *Oct4* and *Klf4* plus 250 μl of *Sox2* or *EIA* filtered viral-containing supernatants plus 6 μg/ml protamine sulfate. Finally, the supernatant was replaced by ESC medium 48 h after transduction. Each reprogramming experiment was performed at least three times. Alkaline phosphatase staining, immunocytochemistry, methylation analysis, *in vitro* differentiation, teratoma formation and morula aggregation have been performed as previously described^18^. Antibodies used for immunocytochemistry are listed in Supporting Information Table S1. Primers are listed in Supporting Information Table S2.

### RNA extraction, cDNA synthesis, and quantitative real-time PCR (qRT-PCR)

RNA was extracted using the QIAGEN RNeasy Mini Kit (QIAGEN), and cDNA synthesis was performed using the High-Capacity cDNA Reverse Transcription Kit (Applied Biosystems). qRT-PCR was carried out using iTaq SYBR Green Supermix with ROX (Bio-Rad). Data was plotted using the delta delta Ct algorithm, 2^(−**∆∆**Ct)^. Primers are listed in Supplementary Table S2.

### DNA extraction and genomic PCR

Genomic DNA was extracted using the QIAamp DNA mini kit (QIAGEN). PCR was performed using *Taq polymerase* (NEB) following the manufacturer’s protocol. Primers are listed in Supplementary Table S2.

### Co-immunoprecipitation (Co-IP)

293T cells were transfected with expression vectors encoding C-terminal 3x FLAG-, 3x Myc- or V5-tagged Oct4, Sox2, E1A or Nanog. 24 h after transfection, cells were harvested and lysed in lysis buffer [50 mM Tris-HCl (pH 7.4), 0.1% IGEPAL CA-630, 5 mM EDTA, 150 mM NaCl] followed by a brief sonication using Bioruptor (Diagenode). Lysates were incubated overnight with anti-FLAG antibody bound to Protein G Dynabeads. After washing three times with wash buffer [50 mM Tris-HCl (pH 7.4), 0.1% IGEPAL CA-630, 5 mM EDTA, 125 mM NaCl], immunoprecipitates were eluted by incubation with 200 μg/ml 3× FLAG peptide for 1 h at 4 °C. Immunoprecipitates and whole cell lysates were subjected to SDS-PAGE and immunoblotted with antibodies against FLAG, Myc and V5. Antibodies used for Co-IP are listed in Supplementary Table S1.

### Luciferase reporter assay

293T cells were transfected with pGL4.75[hRluc/CMV] together with the reporter constructs 6W-37tk-luc, which drives the luciferase under the control of the canonical binding sequence for *POU* factors (the ATGCAAAT sequence repeated six consecutive times)^19^, or with ptk-luc, which was used as a control. The total amount of DNA was maintained constant by adding empty vector. Twenty-four hours after transfection, luciferase activities were measured using Dual-Luciferase Reporter Assay System (Promega). The relative luciferase activity (luc/hRluc) was further normalized to that of empty vector control in each reporter.

### *Sox2*-null ESCs self-renewal assay

*Sox2, Oct4*, and *E1A* were cloned in the hygromycin-resistant PiggyBac plasmid pPBCAG-cHA-IH, a modification of the previously reported pGG131 plasmid that contains a hygromycin selection cassette^20^. 450 ng of the PiggyBac coding for *Sox2, Oct4*, and *E1A* together with 150 ng of pCAG-PBase plasmid were co-transfected into 5 × 10^4^ *Sox2*-null ESCs^5^ using Lipofectamine 2000. Doxycycline (1 μg/ml) was added to the medium 5 h after transfection. Next, cells were transferred onto feeder cells, and selected with 300 μg/ml hygromycin for 1 week. Finally, AP was performed to quantify the number of surviving ESC colonies.

## Additional Information

**How to cite this article**: Marthaler, A. G. *et al*. Enhanced OCT4 transcriptional activity substitutes for exogenous SOX2 in cellular reprogramming. *Sci. Rep.* **6**, 19415; doi: 10.1038/srep19415 (2016).

## Supplementary Material

Supplementary Information
