# Supplementary material for: Enhanced OCT4 transcriptional activity substitutes for exogenous SOX2 in cellular reprogramming
Source: Sci Rep. 2016 Jan 14;6:19415. doi: 10.1038/srep19415 (PMC4725906; doi:10.1038/srep19415)
Supplement: Supplementary Information [file srep19415-s1.doc]

**Supporting Information**

**Enhanced OCT4 transcriptional activity substitutes for exogenous SOX2 in cellular reprogramming**

**Adele G. Marthaler^1^, Kenjiro Adachi^1^, Ulf Tiemann^2^, Guangming Wu^1^, Davood Sabour^2,3^, Sergiy Velychko^1^, Ingo Kleiter^4^, Hans R. Schöler^1,5*^ and Natalia Tapia^1,2*^**

**Supporting Information Table 1.** List of primary and secondary antibodies.

| **Primary antibodies** | **Source** | **Dilution** |
| --- | --- | --- |
| Anti-OCT4 | Santa Cruz, sc-9081 | 1:200 |
| Anti-NANOG | Cosmo Bio Co., REC-RCAB0002P-F | 1:200 |
| Anti-SSEA1 | Millipore, MC-90230 | 1:100 |
| Anti-TUBB3 | Sigma, T8660 | 1:2,000 |
| Anti-SOX17 | R&D Systems, AF1924 | 1:100 |
| Anti-ACTA2 | Dako, M0851 | 1:200 |

| **Secondary antibodies** | **Source** | **Dilution** |
| --- | --- | --- |
| Goat anti-rabbit | Invitrogen, A-11011 | 1:2,000 |
| Rabbit anti-mouse | Invitrogen, A-11061 | 1:2,000 |
| Rabbit anti-goat | Invitrogen, A-11079 | 1:2,000 |

| **Co-IP antibodies** | **Source** |
| --- | --- |
| Anti-FLAG | Sigma, F1804 |
| Anti-Myc Tag | Millipore, 05-419 |
| Anti-V5 | Sigma, V8012 |
| OCT4 | Santa Cruz, sc-5279 |
| SOX2 | Neuromics, GT15098 |

**Supporting Information Table S2.** List of primers used for quantitative real-time PCR, genomic PCR and bisulfite methylation analysis.

| **Quantitative real-time and genomic PCR primers** | |
| --- | --- |
| *Actb* forward | ACTGCCGCATCCTCTTCCTC |
| *Actb* reverse | CCGCTCGTTGCCAATAGTGA |
| *Gapdh* forward | CCAATGTGTCCGTCGTGGAT |
| *Gapdh* reverse | TGCCTGCTTCACCACCTTCT |
| GFP forward * | GGAAAAGAATTGGTATCCAC |
| GFP reverse * | GATTATAACAGCTGGGTTGGC |
| *Hprt* forward | CTGGTGAAAAGGACCTCTCGAA |
| *Hprt* reverse | CTGAAGTACTCATTATAGTCAAGGGCAT |
| *Klf4* forward | TGTGTCGGAGGAAGAGGAAGC |
| *Klf4* reverse | ACGACTCACCAAGCACCATCA |
| *Myc* forward | CTGCGTGACCAGATCCCTGA |
| *Myc* reverse | GCTTGTGCTCGTCTGCTTGAA |
| *Nanog* forward | GAACGGCCAGCCTTGGAAT |
| *Nanog* reverse | GCAACTGTACGTAAGGCTGCAGAA |
| *Oct4* forward | TGTTCCCGTCACTGCTCTGG |
| *Oct4* reverse | TTGCCTTGGCTCACAGCATC |
| pMX-*E1A* forward * | CGGTCCTTCTAACACACCTCCTG |
| pMX-*E1A* reverse * | CCACCAACTCTCACGGCAAC |
| pMX-*Klf4* forward * | GATCCCAGTGTGGTGGTACGG |
| pMX-*Klf4* reverse * | GTGGAGAAGGACGGGAGCAG |
| pMX-*Myc* forward | GATCCCAGTGTGGTGGTACGG |
| pMX-*Myc* reverse | TCGAGGTCATAGTTCCTGTTGGTG |
| pMX-*Oct4* forward * | GATCCCAGTGTGGTGGTACGG |
| pMX-*Oct4* reverse * | GGCGAAGTCTGAAGCCAGGT |
| pMX-*Sox2* forward * | GATCCCAGTGTGGTGGTACGG |
| pMX-*Sox2* reverse * | GGCTTCAGCTCCGTCTCCAT |
| *Rpl37a* forward | ACTTGCTCCTTCTGTGGCAAGAC |
| *Rpl37a* reverse | TTCATGCAGGAACCACAGTGC |
| *Sox2* forward | AACTTTTGTCCGAGACCGAGAA |
| *Sox2* reverse | CCTCCGGGAAGCGTGTACT |

* primer used for both genomic and qRT-PCR.

| **Bisulfite methylation analysis primers** | |
| --- | --- |
| *Oct4* promoter 1^st^ forward | TTTGTTTTTTTATTTATTTAGGGGG |
| *Oct4* promoter 1^st^ reverse | ATCCCCAATACCTCTAAACCTAATC |
| *Oct4* promoter 2^nd^ forward | GGGTTAGAGGTTAAGGTTAGAGGG |
| *Oct4* promoter 2^nd^ reverse | CCCCCACCTAATAAAAATAAAAAAA |
